# Supplementary material for: Distinct Conformations of Mirabegron Determined by MicroED
Source: Adv Sci (Weinh). 2023 Oct 17;10(34):2304476. doi: 10.1002/advs.202304476 (PMC10700164; doi:10.1002/advs.202304476)
Supplement: Supplementary file 1 — Supporting Information [file ADVS-10-2304476-s001.pdf]

ADVANCED SCIENCE
Open Access

Supporting Information

for *Adv. Sci.*, DOI 10.1002/advs.202304476

Distinct Conformations of Mirabegron Determined by MicroED

*Jieye Lin, Johan Unge and Tamir Gonen**

Supporting Information

Distinct Conformations of Mirabegron Determined by MicroED

Jieye Lin[1] Johan Unge[1] and Tamir Gonen[1,2,3]*

[1]Department of Biological Chemistry, University of California, Los Angeles, 615 Charles E. Young Drive South, Los Angeles, California 90095, United States

[2] Department of Physiology, University of California, Los Angeles, 615 Charles E. Young Drive South, Los Angeles, California 90095, United States

[3] Howard Hughes Medical Institute, University of California, Los Angeles, Los Angeles, California 90095, United States

* Corresponding Author T.G. tgonen@g.ucla.edu

Methods

Materials.

Mirabegron (2-(2-Amino-1,3-thiazol-4-yl)-*N*-[4-(2-{[(2*R*)-2-hydroxy-2-phenylethyl]amino}ethyl)phenyl]acetamide) was commercially purchased from InvivoChem and used as received without further recrystallization.

Grid preparation.

Sample preparation followed procedure as described previously.[1] One carbon-coated copper grid (400-mesh, 3.05 mm O.D., Ted Pella Inc.) was pretreated with glow-discharge plasma at 15 mA on the negative mode using PELCO easiGlow (Ted Pella Inc.) for 60s. Around 1 mg of powdery compounds were carefully weighed by a Mettler Toledo (XPR225DR) analytical balance and mixed with a grid in a 10 mL scintillation vial. After gently shaking the vial, the grid was removed and clipped using c-ring and autogrid clip (Thermo Fisher) at room temperature.

MicroED data collection.

The clipped grid was loaded in an aligned Thermo Fisher Talos Arctica Cryo-TEM (200 kV, ~0.0251 Å) at 100 K, equipped with a CetaD CMOS camera (4096 × 4096 pixels) and EPUD (Thermo Fisher) software.[1,2] Screening of size- and thickness-suitable microcrystals was done in the imaging mode (LM 210× and SA 3400×). The MicroED data was collected in the diffraction mode with 741 mm diffraction length (calibrated sample-to-detector distance), 70 µm C2 aperture, and a 50 µm selected area (SA) aperture in the parallel beam condition (45.2% C2 intensity) which resulted in a beam size at approximately 1.4 µm. Typical data collection used a constant rotation rate of ~1° per second over an angular wedge of 100° or 120° from -50° to +50° or -60° to +60°, respectively, with 1s exposure time per frame. Crystals selected for MicroED data collection were isolated and calibrated to eucentric height to maintain the crystal inside the beam during the rotation.

MicroED data processing.

The MicroED data was saved in mrc format and converted to smv format using the mrc2smv software (https://cryoem.ucla.edu/microed).[2] The converted frames were indexed and integrated by XDS.[3,4] Three selected datasets with the highest resolution at 0.9 Å were scaled and merged using XSCALE,[4] and intensities were converted to SHELX hkl format using XDSCONV.[4] The merged dataset showed 99.7% overall completeness, which can be *ab initio* solved by SHELXT[5] with a resolution of 1.01 Å. The structure was refined by SHELXL[6] in Shelxle[7] as a graphical interference to yield the final MicroED structure (Figure 1, Table S1 in Supporting Information).

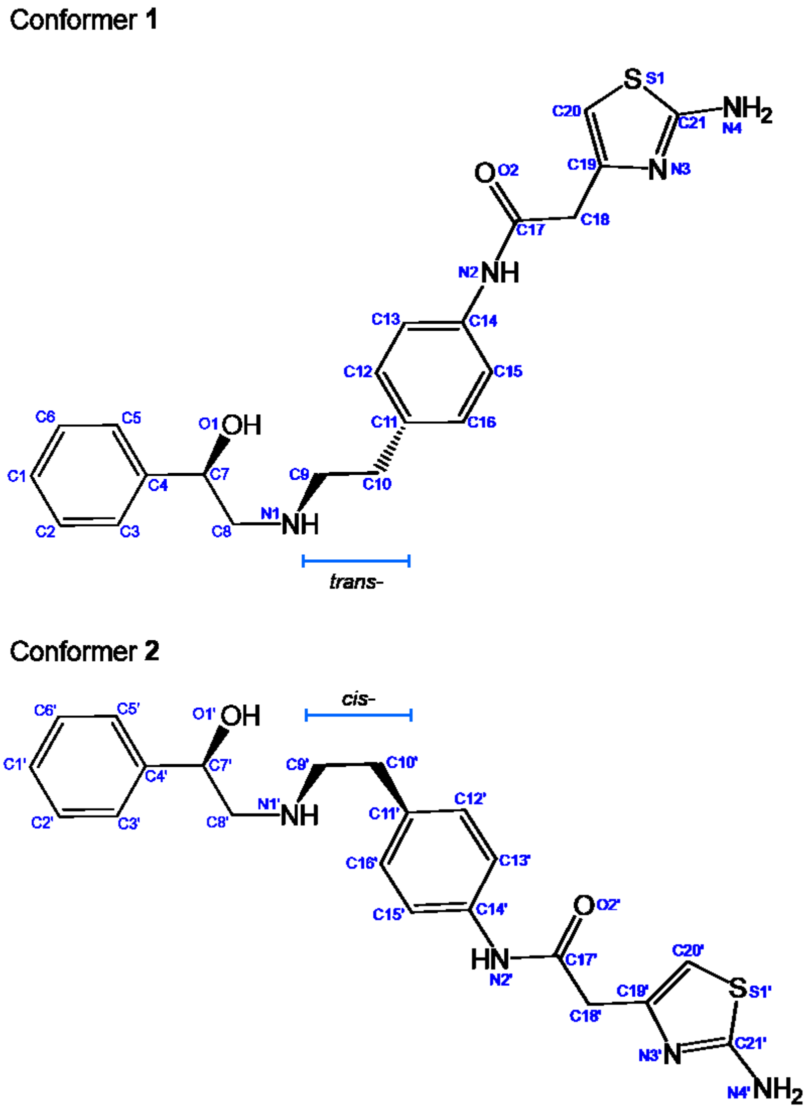

Scheme S1 Chemical notations of Mirabegron. Conformer **1** was labeled with atom type and numbers, conformer **2** was labeled with atom type and primed numbers. Conformations along C9–C10 and C9'–C10' were highlighted, showing the *trans*- and *cis*- form in conformer **1** and **2**, respectively.

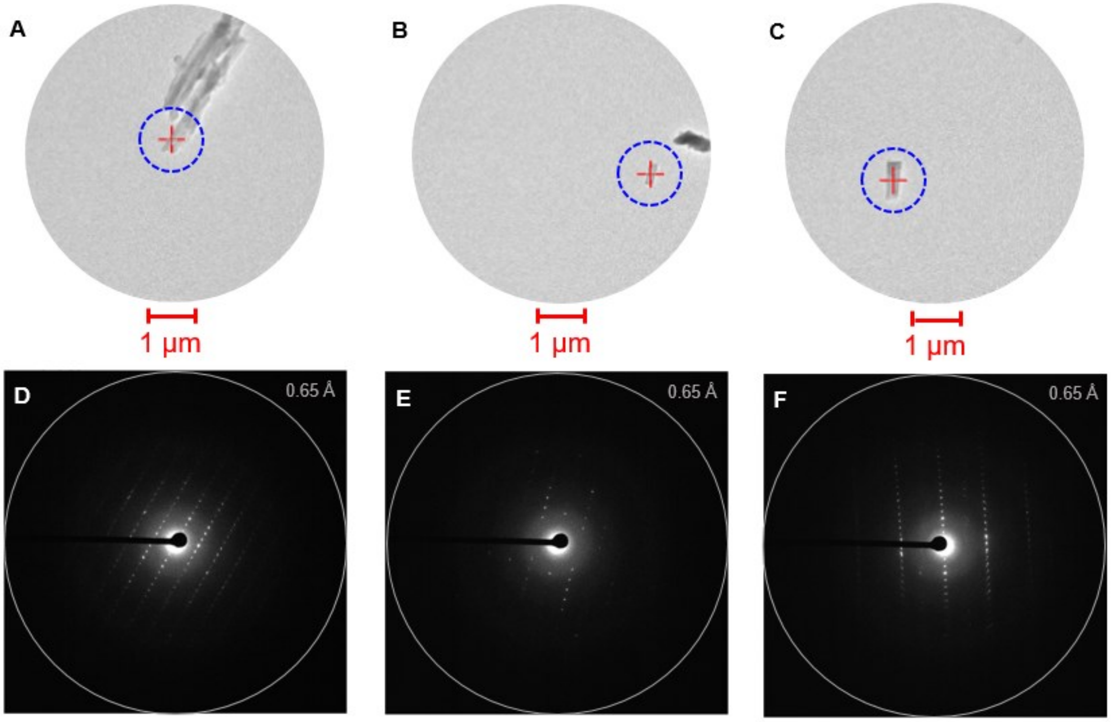

Figure S1 Crystal appearance and diffraction pattern under the TEM. (A-C) Images of items 1-3 under imaging mode (SA 3400×), the diffraction beam size was highlighted in dashed blue circles; (D-F) Diffraction pattern of items 1-3 under diffraction mode (741 mm), the integration edge was colored in grey rings.

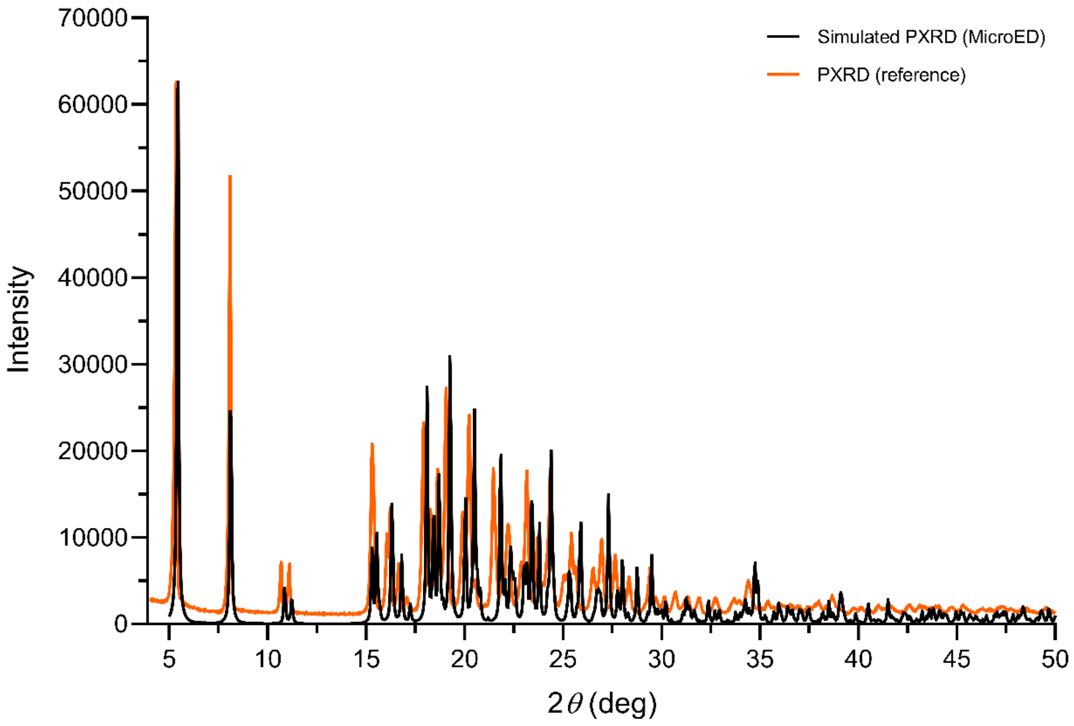

Figure S2 Overlay of literature-reported and simulated PXRD spectra of Mirabegron.[8] Literature-reported PXRD data was colored in orange line; simulated PXRD data was back-calculated from MicroED structure and colored in black line. Intensities were rescaled for comparison.

A

B

C

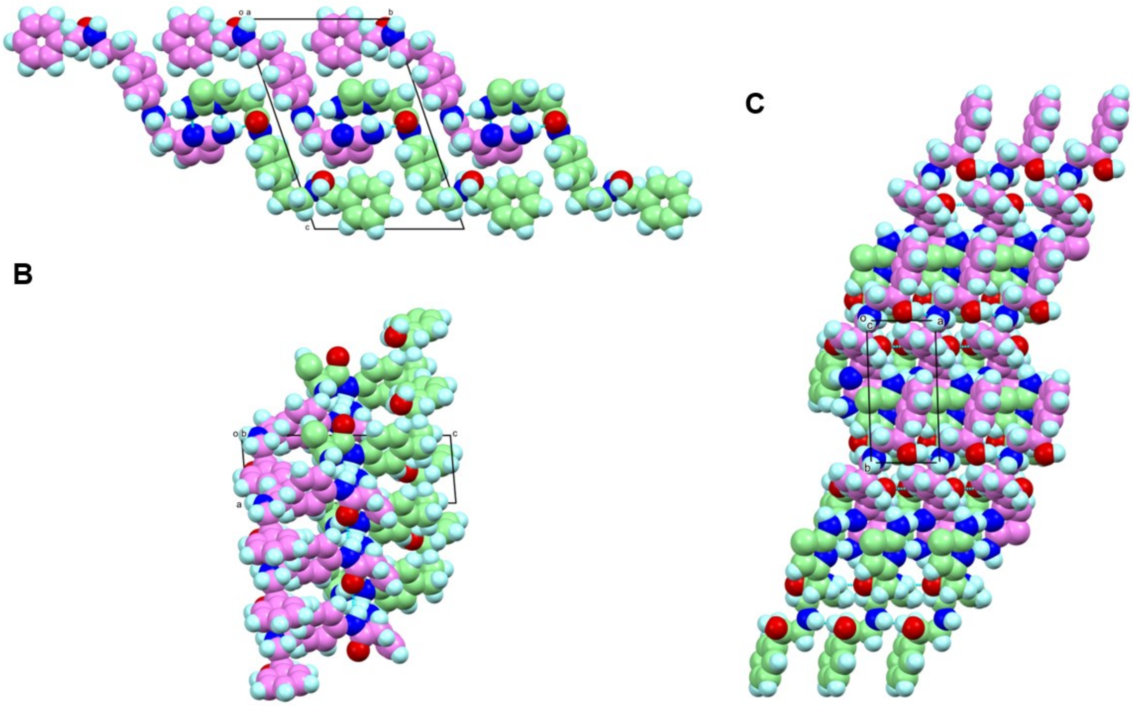

Figure S3 Dense packing of Mirabegron observed in the crystal lattice. (A) viewed along *a* axis; (B) viewed along *b* axis; (C) viewed along *c* axis. Conformer **1** was colored in violet, conformer **2** was colored in light green. The cyan dashed lines represented the hydrogen-bond interactions, with the contact oxygen and nitrogen atoms colored in red and blue, respectively. The hydrogen atoms were colored in cyan.

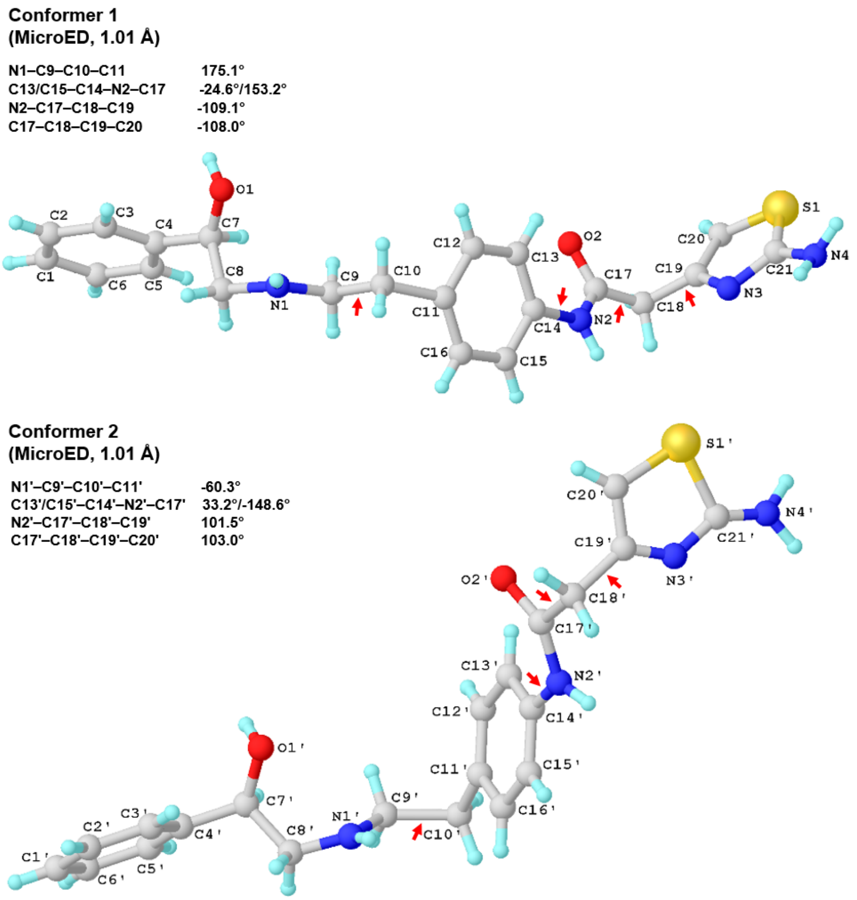

Figure S4 Major structural differences observed in conformer **1** and **2**. The primary torsion differences were highlighted by red arrows. Selected torsion angles were listed for comparison.

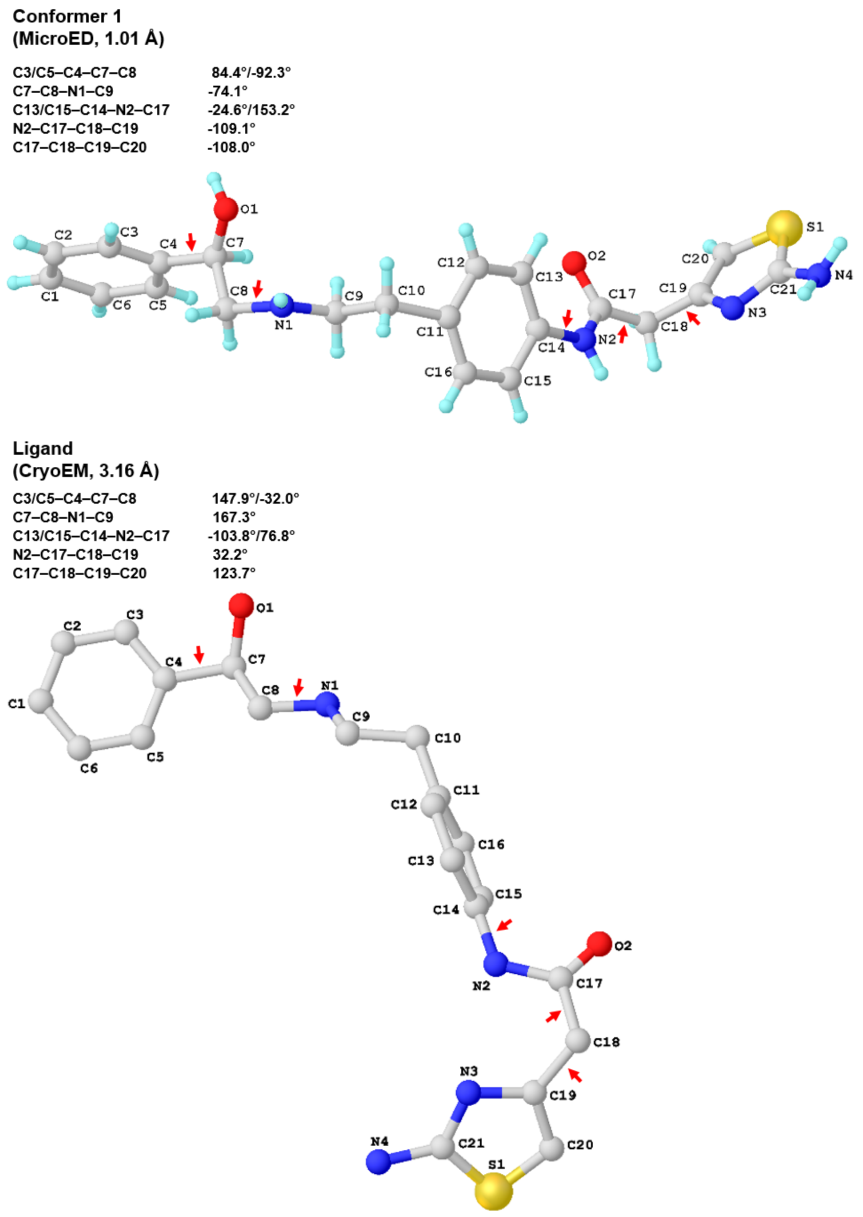

Figure S5 Major structural differences observed in conformer **1** and Cryo-EM structure.[9] The primary torsion differences were highlighted by red arrows. Selected torsion angles were listed for comparison. H atoms were omitted for clarity.

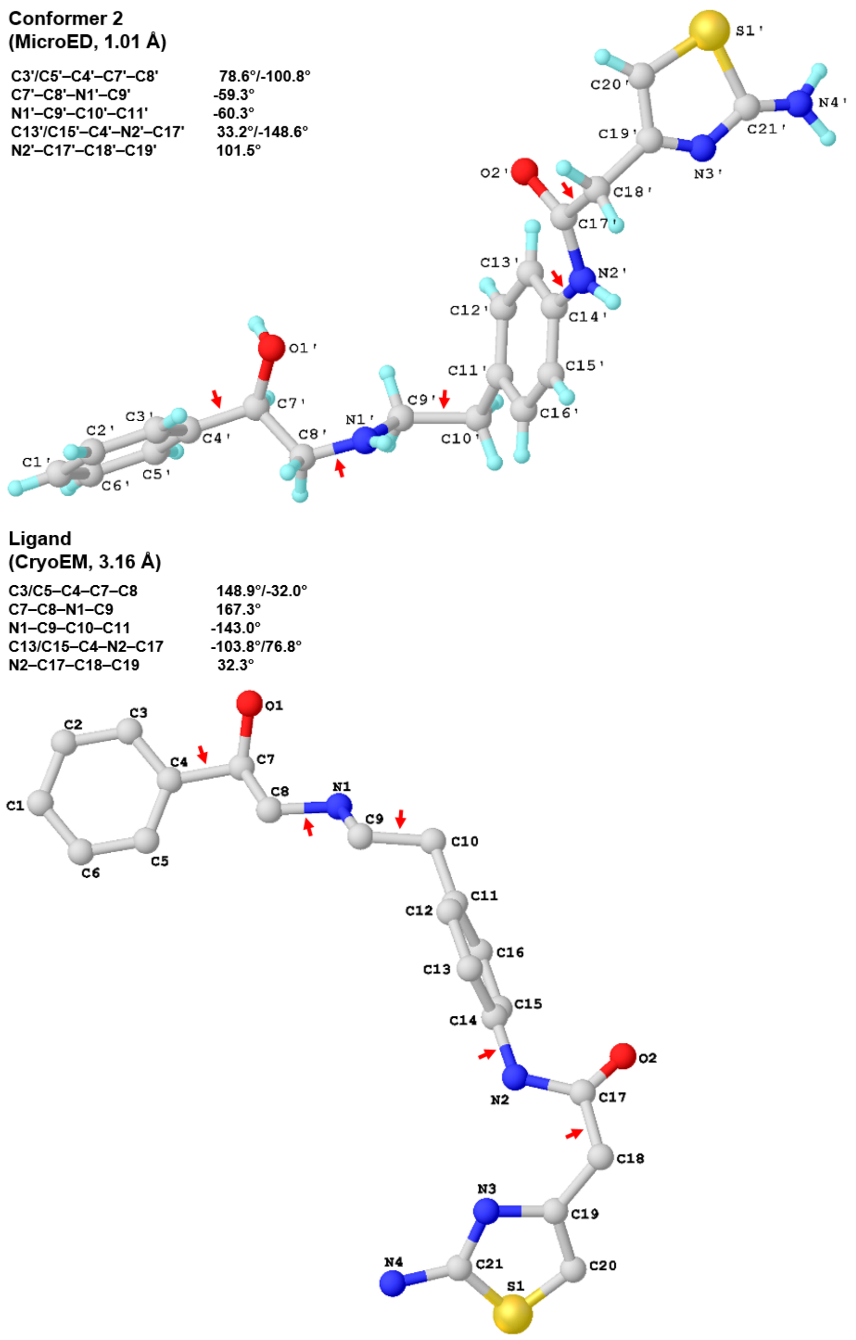

Figure S6 Major structural differences observed in conformer **2** and Cryo-EM structure.[9] The primary torsion differences were highlighted by red arrows. Selected torsion angles were listed for comparison. H atoms were omitted for clarity.

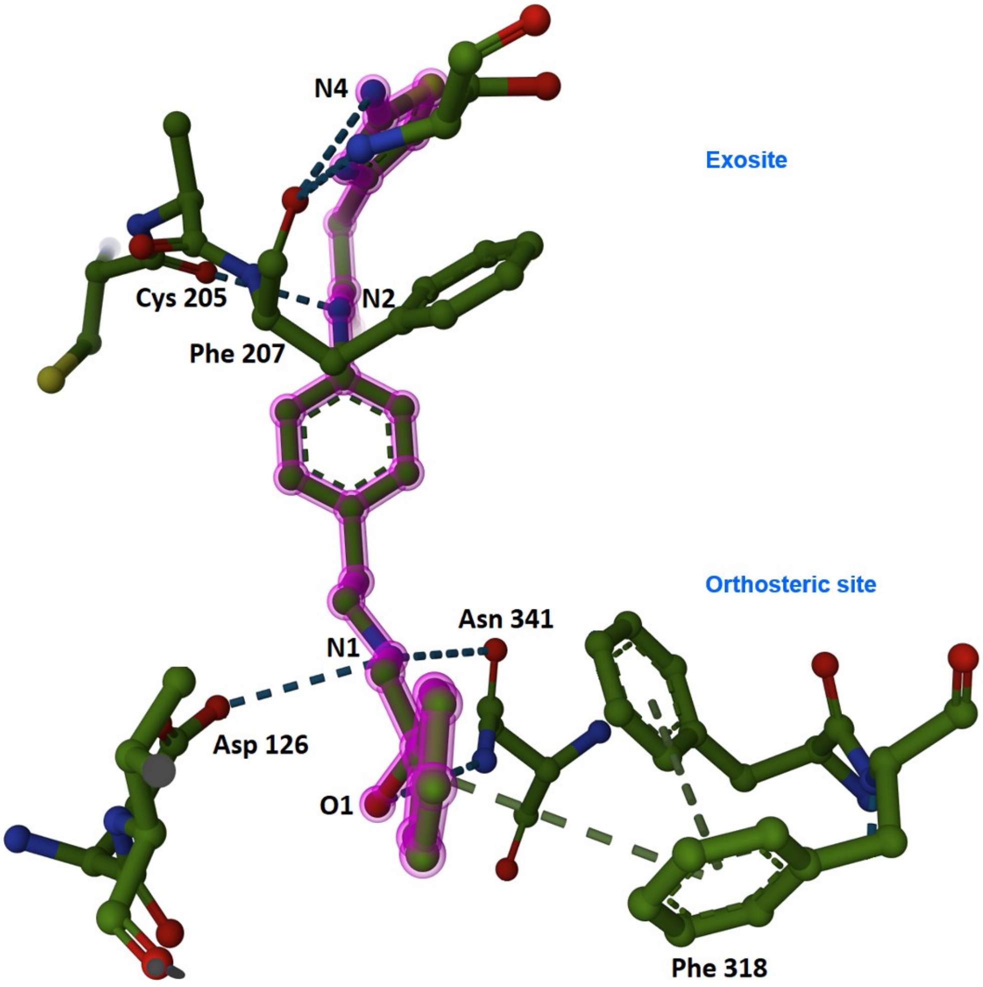

Figure S7 Hydrogen bonding and *van der Waals* interactions between Mirabegron and the active sites of β3AR (PDB entry: 7DH5).[9] Mirabegron was highlighted in violet, and the contact atoms and the residues involved in hydrogen bonding were labeled. H atoms were omitted for clarity.

Table S1 MicroED data statistics of Mirabegron (merged).

Stoichiometric formula	C_{21} H_{24} N_4 O_2 S
Mr	396.50
Temperature (K)	100
Crystal system	Triclinic
Space group	P1
Unit cell lengths (Å)	
a	5.27
b	11.58
c	17.27
Unit cell angles (°)	
α	70.731
β	84.351
γ	86.372
Cell volume (Å^3)	998.51
No. of observed reflections	7518
No. of unique reflections	4014
R_{obs} (%)	18.2
R_{meas} (%)	24.8
I/Sigma	3.09
$CC_{1/2}$	95.5
Resolution (Å)	1.01
Completeness (%)	**99.7**
R_1 (%)	**16.59**
wR_2 (%)	40.17
GooF	1.288

Table S2 MicroED data statistics of three selected items of Mirabegron.

	Item 1	Item 2	Item 3
Space group	P1	P1	P1
Unit cell lengths (Å)			
a	5.27	5.28	5.27
b	11.58	11.41	11.66
c	17.27	17.37	17.18
Unit cell angles (°)			
α	70.731	70.441	71.265
β	84.351	84.632	84.377
γ	86.372	86.375	86.254
No. of observed reflections	6878	6314	4856
No. of unique reflections	3547	3245	2682
R_{obs} (%)	18.3	18.9	14.4
R_{meas} (%)	25.9	26.7	20.4
I/SIGMA	2.39	3.23	3.12
$CC_{1/2}$	97.9	98.4	98.6
Resolution (Å)	0.9	0.9	0.9
Completeness (%)	72.2	66.7	54.2

Table S3 Hydrogen-bond geometry in Mirabegron (Å, °)

**D–H···A**	**D–H**	**H···A**	**D···A**	**D–H···A**
O1–H···N1^{i}	0.825	2.060	2.797	148.59
N2–H···O2^{ii}	0.865	2.514	3.275	147.28
N4–H···O2'^{i}	0.895	2.017	2.907	172.35
N4–H···N3'	0.918	2.294	3.195	167.03
O1'–H···N1'^{ii}	0.839	2.077	2.853	153.61
N2'–H···O2'^{i}	0.867	2.117	2.969	167.58
N4'–H···O2^{ii}	0.928	2.142	3.063	171.81
N4'–H···N3	0.935	2.283	3.086	143.69
Symmetry codes: *(i) x+1, y, z; (ii) x−1, y, z.*				

Reference

1. C. G. Jones, M. W. Martynowycz, J. Hattne, T. J. Fulton, B. M. Stoltz, J. A. Rodriguez, H. M. Nelson, T. Gonen, *ACS Cent. Sci.,* **2018**, *4*, 1587-1592.
2. J. Hattne, M. W. Martynowycz, P. A. Penczek, T. Gonen, *IUCrJ,* **2019**, *6*, 921-926.
3. W. Kabsch, *Acta Crystallogr., Sect D: Biol. Crystallogr.,* **2010**, *66*, 125-132.
4. W. Kabsch, *Acta Crystallogr., Sect D: Biol. Crystallogr.,* **2010**, *66*, 133-144.
5. G. M. Sheldrick, *Acta Crystallogr., Sect. A: Found. Crystallogr. Advances.,* **2015**, *71*, 3-8.
6. G. M. Sheldrick, *Acta Crystallogr., Sect. C: Cryst. Struct. Commun.,* **2015**, *71*, 3-8.
7. C. B. Hübschle, G. M. Sheldrick, B. Dittrich, *J. Appl. Crystallogr.,* **2011**, *44*, 1281-1284.
8. J. H. Q. Mendoza, J. A. Henao, A. P. Aparicio, A. R. R. Bohorquez, *Powder Diffr.,* **2017**, *32*, 290-294.
9. C. Nagiri, K. Kobayashi, A. Tomita, M. Kato, K. Kobayashi, K. Yamashita, T. Nishizawa, A. Inoue, W. Shihoya, O. Nureki, *Mol. Cell.,* **2021**, *81*, 3205-3215.
